# Symbolic Participation or Substantial Learning Behavior? A PSM-Based Comparison Between Honors and Non-Honors Undergraduates from Two Top Elite Universities in China

**DOI:** 10.3390/bs16061037

**Published:** 2026-06-22

**Authors:** Guoxing Xu, Chunmei Hao, Xinyu Kong, Tingting Gao, Mu Liu, Tingzhi Han, Chongguang Wang, Liangliang Wu

**Affiliations:** 1 Faculty of Education, East China Normal University, Shanghai 200062, China; 52274105004@stu.ecnu.edu.cn (X.K.); 52264105004@stu.ecnu.edu.cn (T.G.); 51254105010@stu.ecnu.edu.cn (L.W.); 2 School of Education, Yangzhou University, Yangzhou 225009, China; 007043@yzu.edu.cn; 3 School of Education, Jiangnan University, Wuxi 214122, China; 4 School of Education, Fuyang Normal University, Fuyang 236041, China; wangchongguang@fynu.edu.cn

**Keywords:** honors education, perceived teaching approaches, learning behavior, learning outcomes

## Abstract

Originating in the US and subsequently diffusing across worldwide, honors education has been increasingly adopted in China. A central question is whether honors participation produces substantive changes in students’ learning or functions as symbolic participation. Drawing on samples of senior-year honors (N = 163) and non-honors undergraduates (n = 317) from two top elite universities in China, PSM estimation indicates that honors students do not demonstrate a significant advantage in competence development. However, focusing solely on outcome indicators may obscure the process through which honors education operates. On the one hand, PSM results also showed that honors students were more likely to engage in deep learning behavior. On the other hand, regression revealed that after adding the university as moderator, the significant effect of honors participation disappeared, while the roles of teaching and learning remained consistently stable. Moderated chain mediation analyses further indicated that the association between honors participation and competence development was primarily linked to student-centered teaching practices and deep learning engagement, and that this pathway varied across the two universities. Overall, the findings suggest that the benefits of honors education may derive less from honors affiliation itself and more from the substantive learning experiences fostered within honors contexts. These findings provide empirical support for reforms that place greater emphasis on learning processes and competence development within honors education.

## 1. Introduction

Amid the ongoing massification and growing diversification of student populations in global higher education systems, countries are actively redesigning undergraduate education to enhance talent development and promote academic excellence. Honors education has emerged as a widely adopted institutional response across higher education systems. Existing studies generally demonstrate that honors programs systematically strengthen curriculum rigor, faculty–student interaction, academic culture, and resource support, thereby generating substantial gains in students’ academic abilities, research readiness, and learning experiences (Rinn & Plucker, 2019).

In recent years, driven by China’s “Double First-Class” initiative and national talent strategies, universities have gradually developed a system of honors education represented by programs such as the Top-Notch Talent Training Program, Honors Colleges and Elite Colleges (Shen, 2022). Longitudinal studies indicate that these programs help promote students’ critical thinking, research capability, and academic self-efficacy (Yu et al., 2024). However, does honors education genuinely transform how students learn, or does it function primarily as symbolic participation embedded in selective admission? This concern points to the distinction between symbolic participation and substantial learning behavior, a distinction that remains undertheorized and insufficiently operationalized in existing research. Honors participation can be understood as a mechanism of symbolic capital accumulation (Bourdieu, 2018), whereby program membership signals academic distinction while largely reproducing pre-existing dispositions and advantages. Participation may carry symbolic value even in the absence of observable changes in students’ learning behavior. In contrast, substantial learning behavior refers to observable and sustained transformations in how students engage with learning tasks, particularly shifts toward deeper cognitive engagement, active knowledge construction, and integrative understanding (J. Biggs et al., 2022). If honors programs succeed in reshaping students’ learning behaviors by fostering deep learning, then their value extends beyond elite cultivation and holds implications for mainstream undergraduate teaching (Zhao, 2022).

In fact, the international literature has long recognized that outcome indicators alone cannot explain the value of honors education, nor can they separate selection effects from educational effects. An increasing number of studies argue that the core contribution of honors programs is the construction of a learning environment characterized by higher cognitive demands, more intensive interaction, and stronger academic support, which collectively promote deeper forms of learning engagement (Knox & Heilker, 2023). Gagné’s Differentiated Model of Giftedness and Talent (DMGT 2.0) provides a systematic theoretical articulation of this process (Gagné, 2010). Within this model, learning processes serve as critical mediators of honors effects. Empirical research shows that typical honors features, such as small seminars, undergraduate research, interdisciplinary thematic courses, high-impact practices (HIPs), and mentoring, significantly enhance students’ cognitive engagement, reflective integration, and active inquiry (Miller & Dumford, 2018; Miller et al., 2021).

Although some empirical studies have attempted to examine honors effects within this theoretical framework after controlling for students’ background characteristics, most remain at the level of group comparisons. In the Chinese context, research on the internal mechanisms of honors education is still underdeveloped. Existing studies tend to focus on academic performance patterns, curricular models, or single-process variables, but seldom capture the essential features of teaching and learning (McDaniel et al., 2025). Identifying the substantive and processual effects of honors education in China is thus a pressing issue. Taken together, existing research leaves unanswered whether honors education produces substantive educational effects beyond selection, and if so, through which learning processes such effects unfold. Against this backdrop, this study focuses on undergraduates from Chinese elite universities and seeks to address three key questions: (1) Do honors students differ significantly from non-honors students in their learning behavior and outcomes? (2) After controlling for background characteristics, can honors participation explain these differences? (3) Can the honor effect be mediated by students’ perceived teaching approaches and learning behavior?

By addressing these questions, this study synthesizes the essential characteristics of undergraduate learning through the lens of learning behavior, revealing the process mechanisms through which honors effects unfold, thus moving beyond static group comparisons. This study further contributes by generating actionable, process-oriented evidence to inform the improvement in honors education in China. It also offers empirical grounding for strengthening mainstream undergraduate instruction in the context of higher education massification, and provides the global scholarly community with a fresh lens through which to understand the institutional dynamics and contextual logic of China’s honors education. Additionally, we conduct robustness checks using propensity score matching (PSM), which helps reduce selection bias and supports more rigorous estimation of the statistical associations between honors participation and learning outcomes.

## 2. Literature Review and Hypothesis Development

### 2.1. Theoretical Foundation and Research Framework

A systematic analysis of current honors education systems and practices worldwide reveals a clear trend of transitioning from a giftedness paradigm to a talent development paradigm, and further toward a differentiated instruction paradigm (Dai & Chen, 2013). Naturally, when examining differences in learning outcomes between honors and non-honors students, it is essential not only to consider the honors designation itself but also to clarify the structural differences in the learning contexts and processes experienced by the two groups (Grover & O’Flaherty, 2016). Based on this perspective, the present study examines honors effects through the lens of teaching approaches and learning behavior. In existing higher education effectiveness research, the “Presage-Process-Product” model has become a dominant analytical framework. J. B. Biggs’ (1989) 3P model emphasizes that learning contexts influence learning outcomes through their effects on the learning process. Astin’s (2014) theory further suggests that the core effects of educational contexts are realized through students’ engagement behaviors rather than acting directly on learning outcomes.

**Figure 1:**
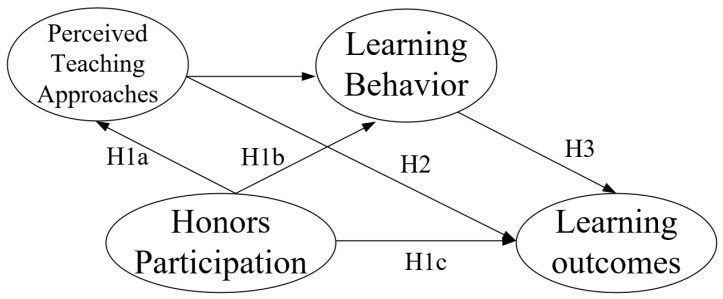
Research Framework.

Accordingly, as illustrated in Figure 1, honors participation is treated as an institutionalized precondition. Teaching approaches and learning behavior are conceptualized as core elements of the honors environment. As noted, teaching approaches enhance students’ cognitive engagement, interaction density, and the demands for deep processing, providing direct stimulation for deep learning. Due to the measurement in this study relies on students’ subjective assessments of instructional practices, teaching approaches are operationalized as perceived teaching approaches to more accurately reflect learners’ interpretation of the learning environment. At the level of internal processing, students in honors contexts are more likely to adopt deep learning behavior, including reflective integration, autonomous inquiry, and metacognitive regulation. Therefore, the sequence from perceived teaching approaches to learning behavior, constitutes a continuous process chain within honors education. In this study, perceived teaching approaches and learning behavior are treated as chain mediators to reveal the process through which honors effects emerge. Finally, learning outcomes represent the manifestation of honors education.

### 2.2. Differences in Learning Outcomes Between Honors and Non-Honors Undergraduate

Rather than simply referring to learning outcomes, this concept represents the tangible results of undergraduates’ professional study and research training, or the key indicators that distinguish academic performance among different students. Scholars typically measure undergraduate learning outcomes through dimensions such as knowledge, competence, and attitudes (Zlatkin-Troitschanskaia et al., 2016). Existing research largely agrees that, compared with non-honors students, honors undergraduates generally demonstrate superior academic performance (Carnicom & Clump, 2004).

Regarding objective indicators of learning outcomes, including GPA, course completion rates, retention, and graduation rates, honors students usually perform better. Multiple longitudinal studies in the United States indicate that honors program participants have significantly higher retention and four-year graduation rates than their non-honors peers (Cosgrove, 2004; Keller & Lacy, 2013). Studies in the Netherlands and other European countries similarly find that honors students are more likely to engage in research projects and pursue advanced academic trajectories (Burke, 2022). More importantly, numerous empirical studies demonstrate that honors students excel in higher-order cognitive skills, including critical thinking, integrative learning, problem-solving, and self-regulation. Large-sample research based on the U.S. National Survey of Student Engagement (NSSE) shows that honors students outperform non-honors students in deep learning strategies, reflective learning, and engagement, indicators commonly recognized as important process markers for cognitive development (Miller & Dumford, 2018). Similar findings have been reported in the United Kingdom, Australia, and New Zealand, highlighting that honors courses promote higher levels of academic preparedness, research ability, and interdisciplinary learning (Evis, 2022; Shipton et al., 2024). However, much of this literature focuses on outcome differentials or isolated engagement indicators, with limited attention to the underlying learning behavior mechanisms through which honors effects are generated. Furthermore, in terms of learning satisfaction and the quality of the learning experience, honors students generally report higher course satisfaction, better faculty–student interaction, stronger academic self-efficacy, and a greater sense of belonging to the institution (Nichols et al., 2016). These affective and identity-related gains in the learning experience are often key reasons why honors education continues to attract students. Yet, existing studies rarely examine whether such advantages persist after rigorously controlling for students’ pre-college characteristics, nor do they sufficiently clarify whether honors participation itself contributes to substantive changes in students’ learning behavior.

### 2.3. The Influence of Honors College or Program

From a development perspective, most studies confirm that honors colleges function as a specialized institutional environment for gifted student development. They encourage undergraduates to pursue excellence as a developmental goal and to engage in corresponding academic behaviors (A. E. P. Cuevas, 2015). The United States, as the birthplace of honors education, implements forms such as research-based learning, interdisciplinary exploration, high-impact practices (HIPs), and learning communities (Stich, 2018). Numerous empirical studies indicate that honors students outperform comparison groups in learning abilities, satisfaction, retention, and graduation rates (Bowman & Culver, 2018). European countries, especially the Netherlands, Germany, and the United Kingdom, have developed distinctive honors programs under the Bologna Process, placing particular emphasis on early research training and interdisciplinary thematic courses. In Australia, the Honors Year directly targets fourth-year undergraduates, providing decisive support for progression to graduate studies (Zeegers & Barron, 2009). Another important perspective concerns elitism, resource allocation, and equity (McMahon et al., 2025). Some studies argue that honors programs may overemphasize academic output, potentially undermining the learning experience (Cognard-Black & Spisak, 2019). Kool’s European study points out that although honors program participants perform better in certain areas, not all indicators of ability, motivation, or creativity show significant improvement (Kool et al., 2017). Others suggest that the benefits associated with honors participation may partly reflect pre-existing advantages linked to academic selection, social background, or cultural capital rather than purely educational effects (Rinn & Plucker, 2019). Nevertheless, even after controlling for these background differences, honors students continue to demonstrate measurable advantages in learning outcomes (Rinn & Plucker, 2019).

Compared with Western contexts, China’s honors education system emphasizes alignment with national talent strategies, aiming to cultivate top-tier innovative talent. Within the broader framework of the Double First-Class initiative, honors colleges often function as institutional platforms through which universities concentrate educational resources, strengthen research-oriented training, and support the development of academically outstanding students. Research by Chinese scholars shows that undergraduates benefit from the external learning inputs provided by these programs, including policy support, challenging courses, and affective support, which significantly enhance learning outcomes (X. Y. Li & Qin, 2019). Other studies also confirm that honors programs have additive advantages for undergraduates’ learning outcomes, such as the development of critical thinking skills (Qin & Lv, 2022). However, honors colleges or programs in Chinese universities have a relatively short implementation history, and most are still in an early, less structured stage, resulting in varied honors effects. Furthermore, there are also some differences in the operational models of honors colleges across different universities. Early Western studies, including a two-year longitudinal study, identified the independent effects of honors courses (Park & Maisto, 1984). Drawing on this approach, Chinese scholars such as T. Z. Han (2022) have explored differences in learning outcomes between honors and regular classes from a comparative perspective, finding that the “joint talent cultivation model” between honors and feeder colleges can increase academic burden and may lead students toward blind academic reverence and inflated self-expectations. These contradictory findings suggest the need for further empirical clarification.

While many studies document outcome differences between honors and non-honors students, fewer specify the instructional and learning processes through which honors participation exerts its effects, particularly after accounting for selection-related advantages. Addressing this gap requires a process-oriented perspective that moves beyond whether honors students perform better, to examine through which pedagogical environments and learning behaviors such differences emerge. From a developmental and comparative perspective, the literature indicates two complementary possibilities. On the one hand, honors programs concentrate institutional resources and design high-challenge, high-support learning environments that plausibly foster superior outcomes. On the other hand, honors cohorts differ systematically from their non-honors peers in selection characteristics such as prior academic preparation and family background, which could account for observed differences. Resolving this ambiguity requires moving beyond group comparisons to specify the intermediate instructional and learning processes through which Honors Participation might operate. In other words, if Honors Participation is associated with systematic differences in outcomes, such differences should be detectable in (a) the instructional environment as perceived by students and (b) students’ adoption of different learning behavior; (c) these process variables, in turn, should account for downstream differences in learning outcomes. This process-oriented logic motivates the hypotheses below.

**H1a.**  *Honors participation is associated with differences in perceived teaching approaches between honors and non-honors undergraduates.*

**H1b.**  *Honors participation is associated with differences in learning behavior between honors and non-honors undergraduates.*

**H1c.**  *Honors participation leads to higher learning outcomes for honors undergraduates compared with non-honors undergraduates.*

### 2.4. Teaching Approaches, Learning Behavior and Undergraduates Learning Outcomes

#### 2.4.1. Teaching Approaches and Undergraduates Learning Behavior

The learning experiences of honors students provide a crucial entry point for exploring the effects of honors participation. However, existing research lacks a high-level synthesis of this learning ecology. In this study, teaching and learning are conceptualized as two interdependent wings of it, interacting with each other to enhance student learning outcomes. J. B. Biggs and Tang (2011) referred to this as a teaching-learning ecosystem. The Teaching and Learning model developed by Trigwell and Prosser (1996) specifically emphasizes how teaching approaches and student learning approaches influence student learning outcomes. Teaching approaches reflect the combination of instructors’ teaching intentions and behavioral strategies, typically categorized into teacher-centered and student-centered approaches (Trigwell et al., 1994). Student learning behavior are closely related to learning motivation and learning strategies, commonly distinguished as deep learning and surface learning (Entwistle, 1997a). Within this framework, teaching approaches do not directly determine learning outcomes but function as proximal contextual cues that invite or constrain particular forms of learning behavior.

Institutional arrangements within honors colleges are often manifested directly in the restructuring of teaching processes (Wolfensberger, 2012). Research suggests that honors colleges stimulate higher-order learning primarily through promoting teaching approaches that are more inquiry-oriented, interactive, and autonomous. For example, Miller et al. (2021) analyzed responses from 1487 instructors across 15 institutions and found that teachers of honors courses were more likely to encourage student engagement in teacher–student interaction, learning strategies, and collaborative learning. These student-centered teaching approaches are regarded as effective pedagogical methods that support students’ problem-based discussions, knowledge construction, and innovative development (van der Zanden et al., 2018). Moreover, students’ perceptions of the instructional environment have been identified as a critical mediator linking institutional design to learning behavior, particularly among high-achieving or academically prepared students (T. Han & Xu, 2025).

#### 2.4.2. Teaching Approaches and Undergraduates Learning Outcomes

Teaching approaches exert a direct and substantive influence on undergraduates’ learning outcomes, as they structure not only the processes through which students engage with learning tasks but also the cognitive, affective, and behavioral dimensions of their academic development. Teacher-centered approaches, characterized by content transmission, unidirectional explanation, and tightly structured learning pathways, have been found to generate relatively constrained learning effects. Empirical research shows that such approaches often limit opportunities for deep cognitive engagement, inquiry-based thinking, and knowledge integration (Kember & Gow, 1994). Students in teacher-centered environments are more likely to adopt surface learning strategies oriented toward reproduction rather than transformation of knowledge, which may suppress higher-order learning outcomes such as problem solving, critical reasoning, and conceptual transfer (Laird et al., 2008).

In contrast, student-centered teaching approaches demonstrate substantial advantages in enhancing learning quality. Multiple studies confirm that student-centered pedagogies lead to deeper cognitive processing, more reflective learning, and higher levels of disciplinary mastery (Gibbs & Coffey, 2004; Baeten et al., 2010). Such approaches stimulate autonomy, foster metacognitive regulation, and promote generative thinking. Research further indicates that these pedagogical environments significantly improve students’ cognitive abilities, creative thinking, and general competencies across disciplines (Adam, 2004; Mintz & Tal, 2013). A large-scale meta-analysis of 225 STEM studies by Freeman et al. (2014) demonstrates that active and student-centered instruction improves academic performance and reduces course failure rates, reinforcing the broad applicability of these findings.

Within honors education, student-centered teaching is not merely a preferred pedagogy but is often institutionally embedded through research-based learning, interdisciplinary seminars, small-group tutorials, and collaborative inquiry projects (Wolfensberger, 2012). However, prior research has largely treated teaching approaches as direct predictors of learning outcomes, without sufficiently considering the relative effectiveness of teacher-centered and student-centered approaches. In response to this inconsistency, the present study builds on the theoretical premise that student-centered teaching better aligns with the developmental goals of higher education, leading to the following hypothesis:

**H2.**  *Student-centered teaching approaches is more strongly associated with overall undergraduate learning outcomes.*

#### 2.4.3. Learning Behavior and Undergraduates Learning Outcomes

Learning behavior represents the internal processing mechanism through which instructional environments translate into measurable learning outcomes. Research grounded in the Student Approaches to Learning (SAL) framework shows that surface learning driven by extrinsic motivation and minimal cognitive investment are linked to fragmented knowledge, poor transferability, and reduced academic achievement (Entwistle, 1997b). Empirical findings across higher education systems indicate that deep learning exert a stronger predictive effect on course grades, higher-order cognitive skills, and overall learning gains than surface strategies (Diseth & Martinsen, 2003; Trigwell & Prosser, 2004). A longitudinal study by Karagiannopoulou and Entwistle (2019) further highlights that deep learning behavior contributes to both academic resilience and sustained growth in advanced competencies.

However, learning outcomes are multidimensional, and the relative advantages of deep versus surface approaches may not be absolute across all learning contexts. In assessment regimes where grades heavily depend on the accurate reproduction of standardized content, surface strategies can produce immediate, measurable academic gains. This observation aligns with the notion that students adapt their learning approaches strategically in response to perceived assessment demands. As Rogoff et al. (1998) argues, learning approaches should not be viewed as static individual traits but rather as repertoires of practice shaped by cultural communities and participation structures. What constitutes “effective” or “valued” learning varies across cultural settings, and the relative weight placed on deep versus surface engagement reflects broader cultural values and institutional practices. In the Chinese context, this complexity is particularly salient. The highly competitive, examination-driven selection system has historically cultivated strong surface-learning dispositions, where memorization and procedural efficiency are prioritized in pursuit of high-stakes test scores (Lam, 2016). Even among honors students, institutional pressures from dual assessment systems and competition for postgraduate recommendations often incentivize surface-oriented strategies, illustrating the tension between the ideal of deep learning and the practical demands of China’s performance-driven higher education landscape (T. Z. Han, 2022).

This study, therefore, does not aim to demonstrate whether deep learning is generally better than surface learning, as this proposition is already well established in the literature. Instead, it asks a comparative question: Does honors participation itself predict learning outcomes more strongly than deep learning behavior? Given the institutional logic outlined above, where honors students are selected for their potential but face persistent pressures that may inhibit deep engagement, it is theoretically expected that deep learning behavior, rather than honors status, functions as the more proximal and powerful predictor of learning outcomes. Accordingly, this study proposes the following hypothesis:

**H3.**  *Deep learning behavior is more strongly associated with overall undergraduate learning outcomes.*

## 3. Methodology

### 3.1. Sampling

This study selected two research-intensive universities that are widely regarded as China’s top elite universities. Data collection was conducted at the end of the first semester of the fourth academic year. This timing was chosen because, by this stage, most students had largely completed their undergraduate coursework and core learning experiences. Honors students were first purposively selected as the focal group, and for each participating honors student, one or two non-honors students were recruited through peer referral to form a matched comparison group, thereby enhancing contextual comparability between groups.

University A is a research-oriented university located in northeastern China, with pronounced strengths in engineering and applied sciences, particularly in fields such as aerospace engineering, telecommunications, computer science, and intelligent manufacturing. University B is a research-oriented university located in eastern China, characterized by balanced development across the sciences and humanities and a strong institutional emphasis on interdisciplinary integration. In terms of scale, University A enrolls over 40,000 students and University B over 30,000 students, with undergraduate populations of approximately 15,000 in each institution and the number of senior-year undergraduates is approximately 3500–4000 per institution. Despite being comparable in scale, the Honors College models at the two institutions exhibit certain differences. At University A, selection for the Honors College is open exclusively to students in the sciences and engineering, and student dormitories is centrally located. At University B, conversely, selection is open to students from all majors, and dormitories is arranged centrally by the university, with Honors students residing alongside their non-Honors peers. Apart from these distinctions, there are no significant differences in the policies of the two Honors Colleges regarding faculty mentorship resources, extracurricular activities, opportunities for direct admission to graduate programs, or scholarship incentives. In both universities, honors students account for no more than 5% of the undergraduate population, corresponding to roughly 150–200 students per cohort. Within this constrained population, the honors students included in this study (N = 163) represent approximately 50% of the total eligible honors student population across the two universities.

After excluding invalid responses, a total of 480 valid questionnaires were obtained, 163 students (34.0%) were honors students. Among them, 167 students (34.8%) were from University A and 313 students (65.2%) from University B. In the sample from University A, all honor students are in natural sciences and engineering; males account for 77.27%, females account for 22.73%, and ages range from 21 to 23. In the sample from University B, the distribution of honor students by discipline is as follows: humanities and social sciences 14.82%, natural sciences and engineering 75.18%. Males account for 70.92%, females account for 29.08%, and ages range from 21 to 23. Based on their basic characteristics and sample structures, certain differences exist between the Honors College operational models of University A and B; consequently, they are treated as moderating variables in this study.

### 3.2. Variable Measurement

In this study, honors participation was defined as a binary variable (0 = non-honors student, 1 = honors student) to indicate whether a student was in an honors education presage. Other independent, dependent, and control variables are as follows:

#### 3.2.1. Perceived Teaching Approaches

The measurement of perceived teaching approaches primarily referred to the Approaches to Teaching Inventory (ATI) developed by Trigwell and Prosser (2004). From the student perspective, the scale was translated and adapted to the context of Chinese higher education. Two bilingual researchers independently translated the original items into Chinese, reconciled differences to produce a preliminary version. A blind bilingual expert back-translated it into English, which was then compared with the original for semantic equivalence. The final version retained 16 items across four dimensions: perceived teacher-centered intentions, perceived teacher-centered strategies, perceived student-centered intentions, and perceived student-centered strategies. Responses were recorded on a five-point Likert scale from 1 (strongly disagree) to 5 (strongly agree), with higher scores reflecting greater perceived teaching approaches.

Cronbach’s alpha was 0.720 for the teacher-centered dimension and 0.815 for the student-centered dimension, indicating acceptable internal consistency for each higher-order factor. Prior to confirmatory factor analysis, an exploratory factor analysis (EFA) was conducted to examine the underlying factor structure of the translated instrument. All items loading on their expected factors and no substantial cross-loadings observed. Subsequently, confirmatory factor analysis (CFA) using Mplus was conducted based on a two-factor model corresponding to teacher-centered and student-centered approaches. The model demonstrated an acceptable fit to the data (χ^2^/df = 2.21, CFI = 0.914, TLI = 0.849, RMSEA = 0.074, SRMR = 0.065). Although the TLI value was slightly below the conventional threshold of 0.90, prior methodological studies have suggested that fit indices such as TLI and CFI are sensitive to model complexity, sample characteristics, and the number of observed variables, particularly in models with multiple factors and translated instruments (Marsh et al., 2004; Hair et al., 2019).

#### 3.2.2. Learning Behavior

The measurement of learning behavior was based on Biggs’ Study Process Questionnaire (R-SPQ-2F). A validated Chinese version developed and tested by prior Chinese scholars (G. Xu, 2021) was adopted in this study to ensure linguistic and cultural appropriateness. The scale includes four dimensions: Deep Motive, Deep Strategy, Surface Motive, and Surface Strategy, with a total of 20 items. Responses were recorded on a five-point Likert scale from 1 (strongly disagree) to 5 (strongly agree), a higher score on a dimension indicates that the student is more likely to adopt that behavior. In line with the theoretical structure of Biggs’ model, the items were aggregated into deep learning and surface learning. Cronbach’s alpha was 0.858 for deep learning and 0.806 for surface learning, indicating good internal consistency. Exploratory factor analysis (EFA) further supported the expected structure. CFA based on this two-factor structure showed acceptable model fit (χ^2^/df = 2.99, CFI = 0.862, TLI = 0.845, RMSEA = 0.064, SRMR = 0.061), providing preliminary evidence for the construct validity of the scale.

#### 3.2.3. Learning Outcomes

The learning outcomes scale was derived from the Chinese College Students Survey (CCSS) conducted by Tsinghua University (Shi et al., 2024). Given that the original questionnaire was designed based on a broad sample of Chinese universities, only minor adjustments were made. The scale included three dimensions: competence development, knowledge acquisition, and learning satisfaction. Competence development and learning satisfaction were measured through self-reported items, including perceived competence gains and satisfaction with both the university and the major. Knowledge acquisition was assessed using indicators such as GPA, major-specific ranking, and scholarship attainment, which were standardized and recoded into a five-point scale to ensure comparability with other dimensions. Responses were recorded on a five-point Likert scale from 1 to 5, with higher scores indicating greater learning outcomes.

Following prior higher education research, learning outcomes were conceptualized as a multidimensional construct. Although knowledge acquisition reflects students’ performance-based outcomes, while competence development and learning satisfaction capture subjective evaluations, these dimensions jointly represent complementary aspects of student learning, including cognitive, behavioral, and affective domains. While some variables were collected using self-reported measures, the inclusion of performance-based indicators and the use of distinct constructs help mitigate the potential influence of common method bias.

Background and control variables included gender, age, family location, annual household income, father’s occupation, mother’s occupation, high school type, high school class type, college entrance examination score, whether the student’s major is a Double First-Class discipline^1^, and major type.

### 3.3. Data Analysis Technique

First, descriptive and comparative analyses were conducted to examine differences in learning behavior and outcomes between honors and non-honors students. Propensity score matching (PSM) was first employed as a quasi-experimental strategy to construct comparable groups of honors and non-honors students based on observed background characteristics (Caliendo & Kopeinig, 2008). Multiple matching algorithms were used, and balance diagnostics confirmed the comparability of the matched samples.

Next, hierarchical regression models were estimated to assess the contributions of honors participation, perceived teaching approaches, and learning behavior to different dimensions of learning outcomes. The model also incorporates university as a moderator.

Finally, to further unpack the underlying process mechanisms, moderated chain mediation analyses were conducted using the PROCESS macro for SPSS 25 (Hayes, 2018). Specifically, this approach was used to test whether perceived teaching approaches and learning behavior function as sequential mediators linking honors participation to learning outcomes.

## 4. Data Analysis and Results

### 4.1. Differences Analysis

As shown in independent-sample *t*-tests (Table 1), using Cohen’s d to quantify the effect size (Goulet-Pelletier & Cousineau, 2018), honors students reported significantly higher levels of perceived teacher-centered approaches (PTAs) compared with non-honors students (M = 3.517 vs. 3.171, t = 6.923, *p* < 0.001, d = 0.651). At the same time, honors students perceived significantly higher levels of student-centered approaches (M = 4.189 vs. 4.002, t = 3.659, *p* < 0.01, d = 0.366). These findings suggest that honors education may not simply replace teacher-centered instruction with student-centered approaches. Rather, honors courses appear to involve both stronger instructional guidance and greater opportunities for student engagement. Regarding learning behavior, the two groups showed no significant difference in surface learning (t = −0.077), suggesting that honors participation (HP) does not automatically reduce the use of surface learning (SL). However, honors students demonstrated significantly higher levels of deep learning (DL) than non-honors students (M = 4.118 vs. 3.552, t = 9.829, *p* < 0.001, d = 0.989). With an effect size close to 1, this represents a large effect, indicating that DL is the most distinctive and salient learning behavior associated with honors education.

The results also show that except for GPA and major-specific ranking, all other indicators had Cohen’s d values greater than 0.2, suggesting that the differences between groups are practically meaningful. Further analysis of the magnitude of differences revealed the following order: learning satisfaction > competence development > knowledge acquisition. Honors students demonstrated pronounced advantages in scholarship attainment, cognitive skills, communication skills, and major satisfaction, indicating that they generally possess higher cognitive levels, better learning experiences, stronger academic identity, and greater emotional engagement. It is noteworthy that while honors and non-honors students showed comparable academic ranks within their majors, the honors students had a significantly lower GPA. This pattern is consistent with the institutional reality of honors colleges as some studies have pointed out (Spisak & Carter Squires, 2016), where honors students are required to take more challenging and rigorously graded honors courses, which tend to suppress absolute GPAs.

### 4.2. Propensity Score Matching Estimation

Since baseline regressions cannot fully address selection bias and confounding effects on the dependent variable, this study further employed PSM to conduct a robustness check on the effect of honors participation. The matching performance of nearest-neighbor matching, kernel matching, and radius matching (0.01) was compared, with radius matching (0.01) showing the best performance. Due to space constraints, only the results concerning university teaching and learning factors are reported.

#### 4.2.1. Balance Test and Common Support Assumption

Table 2 presents the balance test results. After matching (AM), however, the balance improves markedly. The standardized biases for all covariates are substantially reduced, generally falling below the conventional threshold of 10%. Correspondingly, the *t*-tests for group differences become statistically insignificant across all variables (*p* > 0.05), indicating that there are no systematic differences between the treatment and control groups after matching. In addition, the bias reduction rates are consistently high, with most exceeding 70% and several reaching over 90%, further confirming the effectiveness of the matching procedure in eliminating observable differences.

**Figure 2:**
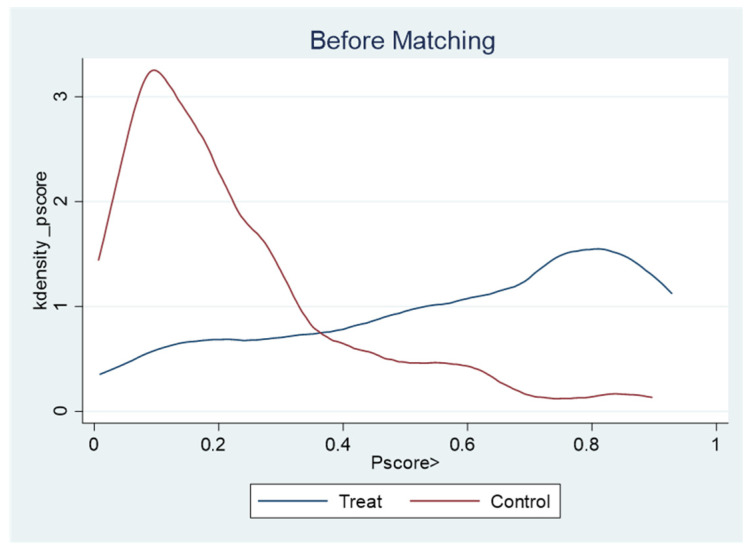
Kernel Density Estimates of Propensity Scores Before Matching.

**Figure 3:**
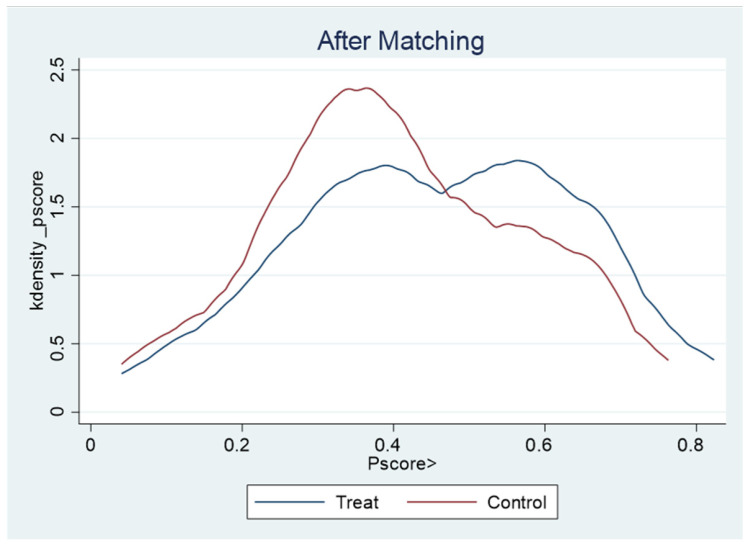
Kernel Density Estimates of Propensity Scores After Matching.

The kernel density plots (Figure 2 and Figure 3) illustrate the propensity score distributions before and after matching. Prior to matching, the treatment and control groups exhibited some differences in their density curves. After matching, the two curves became highly overlapping across most of the propensity score range.

#### 4.2.2. Matching Results

The propensity score-matching results are presented in Table 3. Before matching, honors students reported significantly higher levels of perceived teacher-centered teaching approaches (PTC), perceived students-centered teaching approaches (PSC), and deep learning behavior (DL) compared to non-honors students. No significant difference was found in surface learning behavior (SL). After matching, the differences in PTC approaches, PSC approaches, and SL became statistically insignificant across all three matching methods (nearest neighbor, radius, and kernel matching). However, the difference in DL remained significant: nearest neighbor matching (0.282, *p* < 0.01), radius matching (0.213, *p* < 0.05), and kernel matching (0.239, *p* < 0.01). These results suggest that the observed differences in PTAs and SL between honors and non-honors students are largely driven by endogenous selection into the honors program, whereas the difference in DL persists even after controlling for observable covariates.

The results of propensity score matching are reported in Table 4. The matched estimates show that, after accounting for observable background characteristics, honors students exhibit higher levels of knowledge acquisition (KA) and learning satisfaction (LS) than non-honors students, whereas the difference in competence development (CD) is relatively small (0.077). This pattern suggests that, under the current implementation model, honors programs in China may be more closely associated with differences in knowledge acquisition and academic experiences, while differences in competence development appear less pronounced.

### 4.3. Hierarchical Regression Analysis

Table 5 presents the hierarchical regression results for learning outcomes. For each outcome, models were constructed sequentially by adding blocks of predictors. Specifically, the first model included only honors participation (HP); the second model added the interaction term; the third model only included perceived teaching approaches (PTAs) and learning behavior (LB); and the last model included all independent variables.

HP showed a significant positive effect in some initial models, but these effects became non-significant once PTC, PSC, SL and DL were included. The interaction was significant in early models for KA, but lost significance after SL was added. For CD and LS, the interaction term was generally non-significant or only marginally significant. Thus, the effect of honors status was moderated by university, and this moderation became weak or non-significant when teaching and learning behaviors were controlled.

In contrast, PTAs and LB exhibited relatively stable and robust effects across models and outcomes. For example, SL consistently predicted KA negatively and predicted CD and LS positively. PTC and PSC also showed significant and persistent associations with CD and LS in later models. These patterns indicate that teaching approaches and learning behaviors are more stable and substantive predictors of the outcomes than honors status itself.

### 4.4. Moderated Mediation Analysis

In the HP→PTC→SL→LO model, the direct effect of HP on PTC is not significant (B = 0.157, *p* = 0.053). However, PTC positively predicts LO (B = 0.332, *p* < 0.001), whereas SL negatively predicts LO (B = −0.244, *p* < 0.01). As shown in Table 6, the indirect effect of HP→PTC→LO is significant when UNI = 1 (Effect = 0.114, CI [0.061, 0.179]) but not when UNI = 0. This suggests that, in University B, participation in the honors program is more likely to translate into stronger perceptions of teacher-centered teaching, which, in turn, enhances learning outcomes, whereas this mechanism is weaker in University A. However, the index of moderated mediation for the serial path HP→PTC→SL→LO is not significant (Index = 0.062, CI [−0.002, 0.142]).

In the HP→PTC→DL→LO model, PTC positively predicts DL (B = 0.297, *p* < 0.001), and DL positively predicts LO (B = 0.485, *p* < 0.001). The serial indirect effect HP→PTC→DL→LO is significant when UNI = 1 (Effect = 0.050, CI [0.024, 0.084]) but not when UNI = 0. This indicates that, in University B, teacher-centered teaching is more likely to be transformed into deep learning, thereby improving learning outcomes, whereas this transformation is weaker in University A. Nevertheless, the index of moderated mediation is not significant.

In the HP→PSC→SL→LO model, PSC positively predicts LO (B = 0.431, *p* < 0.001), while SL negatively predicts LO (B = −0.095, *p* < 0.01). The indirect effect HP→PSC→LO is significant when UNI = 1 (Effect = 0.045, CI [0.015, 0.083]) but not when UNI = 0. Importantly, the index of moderated mediation is significant (Index = 0.097, CI [0.006, 0.208]), indicating that UNI significantly moderates the indirect effect of HP on LO via PSC. Specifically, in University B, participation in the honors program more effectively enhances students’ perceptions of student-centered teaching, which, in turn, improves learning outcomes, whereas this mechanism is weak or absent in University A. The serial indirect effect in this model is negligible and not significant.

In the HP→PSC→DL→LO model, the indirect effect HP→PSC→LO is significant when UNI = 1 (Effect = 0.045, CI [0.015, 0.083]), and the index of moderated mediation is also significant (Index = 0.056, CI [0.000, 0.124]). Moreover, the serial indirect effect HP→PSC→DL→LO is significant when UNI = 1 (Effect = 0.038, CI [0.014, 0.065]), with a significant index of moderated mediation. These results indicate that UNI moderates not only the simple mediation via PSC but also the serial mediation through PSC and DL. In University B, student-centered teaching is more likely to be internalized into deep learning and subsequently translated into higher learning outcomes, whereas no significant moderating effect is observed in University A.

Overall, in models centered on PTC, mediation primarily operates through learning approaches, with HP→PTC→DL→LO forming a relatively stable serial mechanism that is not significantly moderated by university type. In contrast, models involving PSC exhibit stronger contextual dependence. In particular, the effect of PSC on learning outcomes via DL is both the strongest and significantly moderated by UNI, indicating high sensitivity to institutional context. This suggests that university type shapes the conditions under which student-centered teaching is implemented, thereby facilitating the transformation from instructional experience to deep learning engagement and ultimately to learning outcomes.

## 5. Discussion

### 5.1. Distinctive Learning Behavior and Differentiated Outcomes of Honors Undergraduates

PSM indicates that honors and non-honors students differ significantly only in deep learning, with effect sizes ranging from 0.213 to 0.282, consistent with prior findings on honors learning engagement (Castro-Johnson & Wang, 2003). Importantly, the results also show that honors and non-honors students do not differ significantly in their adoption of surface learning. Much of the prior research implicitly treats deep and surface learning as opposite ends of a single continuum and assumes that honors students engage less in surface learning. By contrast, the present study shows that honors students’ learning advantage lies not in the absence of surface learning, but in the coexistence of surface and deep approaches, with deep learning being significantly intensified. This suggests that honors participation is associated with a conditional learning enhancement mechanism rather than a categorical shift away from surface learning. Such a pattern reflects an environment that encourages reflective integration and autonomous inquiry while still requiring students to navigate efficiency-oriented academic demands (Litster & Roberts, 2011).

In terms of learning outcomes, descriptive comparisons suggest that honors students demonstrate advantages in knowledge, competence, and learning satisfaction (Scager et al., 2012). However, after controlling for selection effects using PSM, the advantage in competence development becomes statistically non-significant, indicating that observed differences in raw comparisons may be partially attributable to pre-existing student characteristics rather than program effects. By contrast, no significant difference is found in GPA. This may be explained by the norm-referenced grading system in highly selective Chinese elite universities, where performance compression reduces observable variation across students despite differences in learning processes (Hartleroad, 2005). In addition, honors education in these institutions emphasizes access to enriched academic resources and research-oriented experiences rather than differentiated grading mechanisms, which may further weaken the alignment between learning processes and GPA outcomes (Healey et al., 2014; J. Li, 2025).

### 5.2. Honors Participation Is Not Independently Associated with Competence Development

The findings suggest that the association between honors education and different dimensions of learning outcomes is uneven, exhibiting a gradient structure: learning satisfaction shows the strongest positive association, knowledge acquisition is moderate, and competence is weakest. However, after adding the interaction term, the significant effect of honors participation disappears. Crucially, hierarchical regression confirm that the effects of perceived teaching approaches and learning behavior remain consistently stable before and after adding the interaction term. Specifically, the stronger the student-centered teaching and deep learning, the stronger the competence development; the effects on knowledge acquisition and learning satisfaction are opposite: knowledge acquisition is only associated with surface learning, while learning satisfaction is only unrelated to surface learning.

This finding encourages a move away from simplistic claims of universal honors advantages toward a more differentiated understanding of honors effectiveness. Within the Chinese higher education system, honors colleges are primarily designed as institutional arrangements that provide selected students with privileged access to academic resources, research opportunities, small-class instruction, and intensive faculty support (Campbell, 2005; L. Xu & Yan, 2025). In this sense, honors participation appears to provide students with access to distinctive learning opportunities, although participation itself is not independently associated with competence development once learning processes are taken into account. The data suggest that competence growth is more likely to be associated with student-centered teaching practices, sustained deep learning engagement, and continuous cognitive challenge (Keller & Lacy, 2013).

In addition, the significant moderating role of the institutional variable (HP*Uni) suggests that different honors college modes may lead to different outcomes. Specifically, after including the interaction term, the main effect of honors participation became non-significant, and the interaction term was significant only in the knowledge acquisition model. This result highlights the importance of institutional context rather than the superiority of any particular honors model. One possible explanation is that honors programs may differ in organizational structure, curriculum design, faculty involvement, and resource allocation, and these differences may shape how students experience honors education. What appears most important is not the formal structure itself, but whether the institutional environment successfully supports meaningful engagement in learning (A. Cuevas et al., 2017). Future research should therefore pay closer attention to how different honors models organize teaching, learning opportunities, and academic support, and how these institutional arrangements influence student development.

### 5.3. Moderated Chain Mediation of Perceived Teaching Approaches and Learning Behavior

The moderated chain mediation analysis provides a critical extension to existing research by demonstrating that the association between honors participation and learning outcomes is not automatic, but conditional upon a sequence of pedagogical and behavioral variables. Honors participation itself does not constitute a direct causal pathway to learning outcomes; rather, it is associated with amplified instructional signals that in turn, relate to students’ learning behavior. This finding refines the explanatory logic of the 3P learning model (J. B. Biggs, 1989) and Astin’s (2014) involvement theory by situating honors education as a contextual mechanism in which instructional experiences are translated into learning engagement.

Moreover, the model also reveals important asymmetries. For models centered on PTC, mediation operates mainly through learning approaches, with a stable serial mechanism that is not significantly moderated by university type. By contrast, the indirect effect of PSC on learning outcomes via deep learning is both the strongest pathway and significantly moderated by the university variable, indicating high sensitivity to institutional context. This means that university type shapes the conditions under which student-centered teaching is implemented, thereby facilitating the transformation from instructional experience to deep learning engagement and ultimately to learning outcomes. In practical terms, students in University B exhibited a stronger indirect pathway linking perceived student-centered teaching, deep learning, and learning outcomes. The present study cannot determine which specific institutional characteristics account for this difference.

Notably, the PTC→DL pathway slightly exceeds the PSC→DL pathway. This finding challenges the assumption that student-centered teaching is universally superior. In Chinese honors education, where curricula are characterized by high cognitive density and strong academic expectations, teacher-centered instruction may function as an effective scaffold for deep learning. Advanced seminars, frontier knowledge lectures, and close faculty guidance which are hallmarks of honors curricula in top Chinese universities can effectively stimulate deep cognitive processing when students are academically prepared and institutionally selected (Gehrtz et al., 2022). From this perspective, teacher-centered and student-centered approaches should not be viewed as competing pedagogical models but as complementary mechanisms that jointly facilitate deep learning. The identified chain mediation structure therefore helps explain why honors participation does not automatically translate into superior learning outcomes and highlights the role of teaching practices and learning engagement in shaping student development (Hamman et al., 2000).

## 6. Conclusions and Implications

A persistent question in prior research concerns whether honors participation represents merely a form of symbolic participation or whether it is associated with substantive changes in students’ learning processes. Using data from two Chinese elite universities and applying propensity score matching, hierarchical regression alongside moderated chain mediation analysis, this study provides a nuanced answer to this debate. At the outcome level, after controlling for observed covariates through PSM, honors participation does not show a statistically significant advantage in competence development. At the behavioral level, honors students exhibit significantly higher engagement in deep learning, and deep learning appears to be a key mechanism through which honors participation is associated with learning benefits. Furthermore, moderated mediation analysis revealed that students in University B exhibited a stronger indirect pathway linking student-centered teaching, deep learning, and learning outcomes. In this sense, honors participation operates more as a contextual catalyst whose strength appears to depend on contextual conditions surrounding teaching and learning. This interpretation challenges outcome-centered approaches that evaluate honors education primarily through competence indicators alone, and contributes a context-sensitive perspective to the international honors education literature.

### 6.1. Reorient Honors Policy Toward Explicit Competence Development Goals

The findings suggest that, in the two honors programs examined, participation is associated more consistently with differences in learning experiences. As a result, honors participation is observed to serve primarily as a gateway into a high-resource learning environment, without guaranteeing corresponding advantages in students’ competencies. A key policy implication is that honors reform should move beyond a result-centered and resource-driven logic toward a process-oriented learning oriented toward competence development. This shift is broadly aligned with Wolfensberger’s (2012) conceptualization of honors pedagogy, which emphasizes the enhancement in academic competence as a central dimension of honors education. At both national and institutional levels, honors programs need to articulate clear developmental objectives that extend beyond GPA and ranking, particularly in domains such as higher-order cognition, academic communication, integrative problem solving, and research capability. These objectives should be embedded in program design and evaluation systems, thereby shifting policy attention from who enters honors programs to what students are expected to develop through participation. International experience suggests that honors programs are most effective when their quality is assessed in terms of students’ learning trajectories and developmental gains rather than selection intensity alone (Kilgo et al., 2015). Aligning Chinese honors policy with competence-based accountability would help ensure that honors education is evaluated not only by student selection and resource allocation, but also by evidence of student development.

### 6.2. Activate Honors Effects Through Instructional Design and Learning Regulation

The findings suggest that the association between honors participation and competence development is more likely to emerge when supportive instructional practices and deep learning engagement are present. Access to enriched educational opportunities alone does not necessarily translate into developmental gains; what matters is how resources are pedagogically activated (Bušljeta, 2013). At the program level, honors curricula should prioritize instructional environments that combine high academic challenge with structured cognitive support. In the Chinese context, teacher-led, research-oriented instruction plays a critical role in scaffolding deep learning when it emphasizes conceptual integration, intellectual rigor, and sustained inquiry. Honors programs should strategically integrate both to create demanding yet supportive learning ecologies. Equally important is the redesign of assessment and incentive systems for students learning. Existing research suggests that under strong institutional pressures tied to GPA, ranking, and postgraduate recommendation, even honors students may adopt conservative learning strategies that dilute competence development (Everaert et al., 2017). Introducing diversified, formative, and process-sensitive assessment practices can lower the perceived risk of deep engagement while preserving academic rigor. From an international perspective, this shift aligns honors education with a broader movement toward learning-centered governance, in which policy influence is exercised through the shaping of teaching and learning processes rather than through student selection alone.

Moreover, the moderated chain mediation model reveals that the effectiveness of student-centered teaching depends significantly on institutional context. Specifically, University B’s honors mode, which admits students from all majors and integrates dormitories with non-honors students, is more conducive to promoting students’ deep learning, and thereby translating instructional experiences into stronger learning outcomes. In contrast, University A’s mode does not exhibit the same moderating effect.

### 6.3. Limitations and Future Research

While PSM enhances comparability between honors and non-honors students by balancing observed selection factors, the present study does not claim full causal identification. Honors participation is treated as an ex post institutional status rather than an experimentally assigned treatment, implying that unobserved confounders may still affect the estimated associations. Future research could address these limitations using longitudinal designs, quasi-experimental approaches, or more explicit modeling of selection into honors programs to better disentangle pre-existing differences from program effects.

Both perceived teaching approaches (PTC/PSC) and learning behavior (SL/DL) were assessed through students’ perceptions, which may be subject to social desirability or recall bias. Self-reported learning outcomes may also not fully capture students’ authentic competence development. Nevertheless, they remain one of the most widely used and methodologically feasible approaches for assessing complex, latent learning constructs at scale in higher education research (Corneille & Gawronski, 2024). Future studies could move beyond single-source data by integrating multi-modal evidence, including classroom observations, assignment-level analytics, digital learning traces, and even experimental task-based assessments.

Although learning outcomes included knowledge gains, ability development, and satisfaction, the analysis revealed that honors experience did not significantly improve ability scores after controlling for learning behavior. This suggests that ability development may require finer-grained measurement or longer-term observation, as competencies such as research skills or critical thinking may only emerge after extended engagement. Future research could refine the operationalization of ability by distinguishing between domain-specific and transferable competencies, and by employing performance-based or longitudinal indicators to capture their gradual formation. In addition, subsequent studies may explore potential mediating and moderating mechanisms, such as learning motivation, epistemic beliefs, or institutional support structures, to better explain under what conditions honors education translates into higher-order competence development.

In addition, the moderated effects identified in this study should be interpreted with caution due to the lack of quantifiable descriptive data on institutional characteristics. Although University A and University B differ in admission scope and dormitory arrangement, these binary descriptors do not capture the full complexity of each honors college’s pedagogical culture, faculty engagement, curricular flexibility, or peer interaction dynamics. Consequently, while we find that University B’s mode appears more conducive to activating student-centered teaching perceptions and deep learning, we cannot definitively attribute this advantage to any specific institutional component. Future research should develop more fine-grained measures of institutional characteristics and examine whether the observed mechanisms generalize beyond elite universities to other types of honors programs and higher education contexts.

## Figures and Tables

**Table 1:** Differences in Learning Outcomes Between Honors and Non-Honors Students.

Variable	Mean ± SD		*t*-Test	Cohen’s d
	Honors Students	Non-Honors Students		
Perceived Teacher-centered Approaches (PTAs)	3.517 ± 0.566	3.171 ± 0.492	6.923 ***	0.651
Perceived Student-centered Approaches (PSAs)	4.189 ± 0.448	4.002 ± 0.569	3.659 **	0.366
Surface Learning (SL)	2.861 ± 0.774	2.867 ± 0.670	−0.077	−0.007
Deep Learning (DL)	4.118 ± 0.486	3.552 ± 0.646	9.829 ***	0.989
GPA	3.979 ± 0.648	4.194 ± 0.605	−3.520 ***	−0.344
Major Grade Ranking	3.620 ± 1.026	3.420 ± 1.024	1.994 *	0.195
Scholarship Award	3.563 ± 1.150	2.726 ± 1.276	7.274 ***	0.675
Competence development	4.041 ± 0.461	3.540 ± 0.718	8.073 ***	0.967
Learning Satisfaction	4.264 ± 0.540	3.457 ± 0.966	9.894 ***	0.942
N	163	317	—	—

Note: * *p* < 0.05, ** *p* < 0.01, *** *p* < 0.001.

**Table 2:** Balance Test of Matched Samples.

Matching Variable	Effect	Mean		Standardized Bias (%)	Reduction (%)	t	*p*
		Treatment	Control				
Gender	BM	1.282	1.440	−33.3	94.9	−3.38	0.001
	AM	1.36	1.352	1.7		0.13	0.895
University	BM	0.865	0.553	73.0	100.0	7.14	0.000
	AM	0.824	0.824	0.0		0.00	1.000
Discipline	BM	1.963	3.106	−102.6	96.5	−10.65	0.000
	AM	2.12	2.08	3.6		0.27	0.788
High School Type	BM	1.589	1.709	−15.6	73.3	−1.57	0.118
	AM	1.608	1.64	−4.2		−0.34	0.733
Class Type	BM	1.080	1.262	−49.7	82.4	−4.80	0.000
	AM	1.096	1.128	−8.7		−0.80	0.424
Entrance Exam (Gaokao) Score	BM	2.380	2.609	−25.7	72.0	−2.79	0.006
	AM	2.352	2.416	−7.2		−0.54	0.592
Hometown	BM	1.755	1.838	−11.4	90.4	−1.19	0.235
	AM	1.744	1.736	1.1		0.08	0.934
Household Income	BM	2.245	1.811	55.2	98.2	5.66	0.000
	AM	2.136	2.128	1.0		0.08	0.936
Father’s Occupation	BM	1.571	1.613	−8.5	23.9	−0.88	0.379
	AM	1.528	1.56	−6.5		−0.51	0.613

**Table 3:** Propensity Score-Matching Results for the PTAs and LB of Honors and Non-Honors Students.

	Honors Students vs. Non-Honors Students			
	PTC	PSC	SL	DL
Before Matching	0.342 *** (0.054)	0.166 *** (0.049)	−0.010 (0.068)	0.556 *** (0.057)
Nearest Neighbor Matching	0.071 (0.096)	0.020 (0.096)	−0.215 (0.122)	0.282 ** (0.117)
Radius Matching (0.01)	0.006 (0.083)	0.013 (0.083)	−0.197 (0.111)	0.213 * (0.095)
Kernel Matching	0.073 (0.080)	0.045 (0.080)	−0.129 (0.108)	0.239 ** (0.094)

Note: * *p* < 0.05, ** *p* < 0.01, *** *p* < 0.001. PTC = Perceived Teacher-Centered teaching approaches; PSC = Perceived Student-Centered teaching approaches; SL = Surface learning behavior; DL = Deep learning behavior.

**Table 4:** Propensity Score-Matching Results for the LO of Honors and Non-Honors Students.

	Honors Students vs. Non-Honors Students		
	KA	CD	LS
Before Matching	0.331 ** (0.088)	0.468 *** (0.061)	0.777 *** (0.082)
Nearest Neighbor Matching	0.517 ** (0.173)	0.227 (0.122)	0.525 ** (0.165)
Radius Matching (0.01)	0.472 ** (0.147)	0.158 (0.101)	0.392 ** (0.138)
Kernel Matching	0.440 ** (0.144)	0.184 (0.010)	0.401 ** (0.136)

Note: ** *p* < 0.01, *** *p* < 0.001. KA = Knowledge Acquisition; CD = Competence Development; LS = Learning Satisfaction.

**Table 5:** Hierarchical Regression Results.

Variable	KA				CD				LS			
	M1	M2	M3	M4	M5	M6	M7	M8	M9	M10	M11	M12
HP	0.282 **	0.191		0.196	0.280 ***	0.231		0.161	0.589 ***	0.313		0.244
Uni	0.106 *	0.009		−0.018	0.109	0.094		0.021	−0.090	−0.15		−0.219
HP*Uni		0.644 **		0.569 *		0.068		−0.069		0.374		0.231
PTC			0.111	0.097			−0.046	−0.052			0.211 **	0.186 *
PSC			0.065	0.069			0.300 ***	0.305 ***			0.345 ***	0.359 ***
SL			−0.150 *	−0.139 *			0.047	0.046			−0.044	−0.040
DL			0.148	0.113			0.543 ***	0.528 ***			0.426 ***	0.402 ***
Others	Controlled											
Cons	4.391 ***	4.216 ***	3.878 ***	3.640 ***	3.718 ***	3.698 ***	2.727 ***	0.418 ***	3.627 ***	3.698 ***	0.150	0.078
F	4.06	4.4	4.02	4.22	2.607	9.74	33.89	29.72	13.36	12.45	21.25	20.83
R^2^	0.08	0.094	0.101	0.120	0.100	0.186	0.486	0.490	0.222	0.226	0.372	0.402

Note: * *p* < 0.05, ** *p* < 0.01, *** *p* < 0.001. HP is coded as a binary variable (0 = non-honors [reference group], 1 = honors). HP = Honors Participation. HP*Uni = University’s honors college model (University A’s model = 0, University B’s model = 1).

**Table 6:** Indirect Effects of honors education on LO (Serial Moderated Mediation).

Path		Uni	Effect Value	S.E.	BC 95% CI	
					Lower 5%	Upper 5%
HP→PTC→SL→LO	HP→PTC→LO	0	0.052	0.027	−0.001	0.107
		1	0.114	0.030	0.061	0.179
	Index (moderated mediation)		0.062	0.037	−0.002	0.142
	HP→SL→LO	-	0.043	0.017	0.011	0.079
	HP→PTC→SL→LO	0	−0.019	0.011	−0.041	0.000
		1	−0.042	0.011	−0.066	0.023
	Index (moderated mediation)		−0.023	0.013	−0.051	0.001
HP→PTC→DL→LO	HP→PTC→LO	0	0.010	0.009	−0.003	0.031
		1	0.023	0.015	−0.005	0.056
	Index (moderated mediation)		0.012	0.011	−0.003	0.040
	HP→DL→LO	-	0.224	0.034	0.160	0.293
	HP→PTC→DL→LO	0	0.023	0.012	−0.001	0.048
		1	0.050	0.016	0.024	0.084
	Index (moderated mediation)		0.027	0.017	−0.002	0.067
HP→PSC→SL→LO	HP→PSC→LO	0	0.018	0.042	−0.108	0.057
		1	0.079	0.027	0.029	0.137
	Index (moderated mediation)		0.097	0.051	0.006	0.208
	HP→SL→LO	-	−0.004	0.007	−0.019	0.010
	HP→PSC→SL→LO	0	−0.001	0.002	−0.007	0.003
		1	0.004	0.002	0.001	0.010
	Index (moderated mediation)		0.005	0.004	0.000	0.014
HP→PSC→DL→LO	HP→PSC→LO	0	−0.010	0.025	−0.062	0.036
		1	0.045	0.017	0.015	0.083
	Index (moderated mediation)		0.056	0.032	0.000	0.124
	HP→DL→LO	-	0.186	0.026	0.138	0.240
	HP→PSC→DL→LO	0	−0.009	0.021	−0.051	0.031
		1	0.038	0.013	0.014	0.065
	Index (moderated mediation)		0.047	0.025	0.000	0.099

## Data Availability

The raw data supporting the conclusions of this article will be made available by the authors on request.

## Note

1 The “Double First-Class” initiative refers to a national higher education development strategy launched by the Chinese government in 2017, aimed at building world-class universities and world-class disciplines. Disciplines included in this initiative receive prioritized policy support and resources.

## Abbreviations

The following abbreviations are used in this manuscript:

HP	Honors Participation
PTC	Perceived Teacher-Centered teaching approaches
PSC	Perceived Student-Centered teaching approaches
SL	Surface learning behavior
DL	Deep learning behavior
KA	Knowledge Acquisition
CD	Competence Development
LS	Learning Satisfaction
